# Stable Across Time and Targets? Test–Retest Reliability of the Emotional Accuracy Test

**DOI:** 10.3390/jintelligence14100240

**Published:** 2026-10-02

**Authors:** Jacob Israelashvili

**Affiliations:** Department of Education, Ariel University, Ariel 4070000, Israel; jacobis@ariel.ac.il

**Keywords:** emotional accuracy, emotion recognition, empathic accuracy, test–retest reliability, naturalistic stimuli

## Abstract

The Emotional Accuracy Test (EAT) assesses performance-based emotional understanding by comparing perceivers’ ratings with targets’ multidimensional self-reports. Prior research supports its convergent and construct validity; the present studies examined test–retest reliability. Study 1 (*N* = 27; mean interval = 25.52 days) repeated the same targets and showed rank-order stability, *r* = .58, 95% CI [.26, .79], and absolute agreement, ICC(A,1) = .59, 95% CI [.20, .80], with no systematic mean-level change. Study 2 (*N* = 62; mean interval = 17.00 days) used different target sets and showed rank-order stability, *r* = .69, 95% CI [.53, .80], and consistency, ICC(C,1) = .65, 95% CI [.49, .76]. Absolute agreement was lower, ICC(A,1) = .52, 95% CI [.37, .63], with higher Time 2 scores. Target set was confounded with occasion and scored composites differed in length (three vs. four videos), so the mean difference cannot be uniquely attributed to occasion. Adjusting for mean rating intensity reduced Study 2’s correlation to .49, a conservative sensitivity estimate because adjustment may also remove accuracy-relevant variance. Together with prior validity evidence, these findings provide additional psychometric support for using the EAT in group-level and correlational research.

## 1. Introduction

Accurately understanding another person’s emotions is central to adaptive social interaction. Yet, many established emotion-recognition tests rely on brief, posed, and prototypical expressions presented outside a meaningful social context. Such tasks offer strong experimental control, but they sample only a restricted portion of the information available when emotions are communicated in everyday life. In natural interactions, emotional understanding depends on integrating facial, vocal, verbal, and contextual information as it unfolds over time (Barrett et al., 2011; Zaki et al., 2009). Advancing the measurement of interpersonal accuracy therefore requires naturalistic, dynamic, and target-referenced paradigms that preserve this complexity (Dawel et al., 2026; Talipski et al., 2026).

The empathic accuracy tradition provided an influential response to this challenge. Rather than defining accuracy according to an externally assigned emotion label, empathic accuracy paradigms compare perceivers’ judgments with targets’ reports of their own internal experiences (Ickes et al., 1990). In the continuous affect-rating paradigm developed by Zaki et al. (2008), for example, perceivers watch targets recount autobiographical experiences and continuously estimate how positively or negatively the target feels. Accuracy is quantified as the correspondence between the perceiver’s and target’s moment-to-moment affect ratings. This approach preserves temporal changes in emotional experience and anchors accuracy in the target’s perspective. At the same time, reducing emotional experience to a single valence dimension leaves unresolved whether perceivers can identify the distinct emotions that compose that experience. Recent evidence of limited correspondence across targets and task formats further suggests that performance in one empathic accuracy context may not straightforwardly reflect a general perceiver ability (Zikic et al., 2025).

The Emotional Accuracy Test (EAT; Israelashvili et al., 2020) builds on this target-referenced tradition while extending it in an important direction. In the EAT, perceivers watch short videos of people describing authentic emotional events and estimate the intensity of multiple emotions experienced by each target. These estimates are compared with the target’s own ratings of the same emotions. Thus, rather than assessing whether perceivers track broad changes in positive and negative affect, the EAT assesses how closely they reproduce a target’s multidimensional emotional profile. Its primary score reflects the inverse absolute discrepancy between perceiver and target ratings across multiple discrete emotions and intensities. This distinction is consequential because recognizing that another person feels negatively is not equivalent to identifying whether that experience involves sadness, fear, anger, shame, or some combination of these emotions.

Evidence accumulated across several studies supports the validity and theoretical usefulness of this approach. EAT performance converges with established emotion-recognition measures based on posed or enacted expressions, including the Reading the Mind in the Eyes Test (Baron-Cohen et al., 2001) and the Geneva Emotion Recognition Test (Schlegel et al., 2014), while using more naturalistic, dynamic, and context-rich stimuli (Israelashvili et al., 2021). Higher emotional accuracy has also been associated with more effective interpersonal communication, including greater linguistic coordination with a conversation target (Israelashvili & Fischer, 2023). Conversely, reduced accuracy has been observed among individuals reporting greater social anxiety and personal distress (Israelashvili et al., 2024). Experimental findings further indicate that emotional understanding can improve following feedback and repeated exposure to the same target (Israelashvili & Perry, 2021), and that training-related gains may generalize to unfamiliar targets under some conditions (Israelashvili et al., 2025).

Together, these findings position the EAT as a measure of both meaningful individual differences and learning-related change. These two uses, however, depend on establishing the temporal stability of its scores. Associations with individual characteristics assume that the EAT can reliably distinguish between perceivers, whereas the interpretation of training effects requires observed change to be distinguished from ordinary fluctuation across measurement occasions (de Vet et al., 2006). Evidence of construct validity or responsiveness to feedback does not, by itself, establish either form of reliability.

Although psychometric work has strengthened several established emotion-recognition instruments (e.g., Schlegel & Scherer, 2016; Wilhelm et al., 2014), behavioral measures are still not routinely evaluated for reliability when used to study individual differences (Parsons et al., 2019). This issue is also relevant to naturalistic tasks, in which scores may depend on the targets, emotional contexts, and scoring procedures sampled. Evidence concerning the temporal stability of EAT scores would therefore provide an important additional basis for interpreting them both as indicators of individual differences and as outcomes in training research.

Direct evidence concerning the test–retest reliability of naturalistic empathic accuracy paradigms remains limited. Kern et al. (2013) administered a continuous valence-tracking task twice, four weeks apart, to clinically stable outpatients with schizophrenia. Scores based on six and nine video clips showed rank-order correlations of .67 and .72, respectively, with little evidence of mean-level practice effects. These findings provide initial evidence that a related naturalistic task can preserve participants’ relative standing over time. However, the healthy comparison group was not retested, and the greater variability observed in the clinical group may have facilitated rank-order reliability. Moreover, Pearson correlations assess whether individuals retain their relative positions, but not whether the same numerical scores are reproduced across sessions. Kern et al. (2013) therefore provided evidence for rank-order stability in a heterogeneous clinical sample, but did not establish absolute-agreement reliability in nonclinical adults.

### The Current Research

The present research provided a preliminary test of whether the Emotional Accuracy Test (EAT) yields temporally stable individual differences in understanding others’ emotions. Test–retest reliability depends partly on the design through which it is assessed. Repeating identical stimuli evaluates whether scores can be reproduced under closely matched conditions, but agreement may be strengthened by memory for the targets or familiarity with their stories. In contrast, using different stimuli provides a more stringent test of whether participants retain their relative standing across targets, although differences in stimulus difficulty may reduce absolute agreement between sessions.

To address these complementary questions, the present research used two test–retest designs. Study 1 examined repeated-stimulus reliability by presenting the same four videos at both sessions. Study 2 examined alternate-form reliability by presenting different target sets at the two sessions. The retest interval was selected to reduce the influence of immediate recall while limiting the likelihood of substantive change in the underlying ability, and to facilitate comparison with Kern et al. (2013), who used a similar interval in a related paradigm. Across studies, the analyses assessed rank-order stability using Pearson’s correlation, absolute agreement using intraclass correlation coefficients, and systematic mean-level change across sessions. This combination distinguishes whether participants retained their relative standing from whether their numerical scores were reproduced over time, because a strong correlation can coexist with a systematic difference between sessions (Koo & Li, 2016).

In Study 2, where the target sets differed, the analyses additionally distinguished consistency from absolute agreement. The consistency coefficient assessed whether participants retained their relative standing across forms, whereas the absolute-agreement coefficient assessed whether the forms also yielded comparable numerical scores. Within-session consistency was examined at the video level in each study. Together, the two designs provide complementary tests concerning the reproducibility of EAT scores under repeated-stimulus conditions and the generalization of individual differences across different naturalistic targets. Based on the two designs, positive rank-order stability was expected in both studies, with stronger absolute agreement when the same stimuli were repeated in Study 1 and potentially lower absolute agreement when different target sets were evaluated in Study 2.

## 2. Method

### 2.1. Participants and Procedure

#### 2.1.1. Study 1: Repeated-Stimulus Design

Participants were recruited voluntarily through students’ social networks as part of a course assignment. Recruitment instructions specified an age criterion of 18 years or older. Of 82 Time 1 response records, 60 contained complete EAT data across all four scored videos. Three respondents with otherwise complete data reported ages below 18 years (16, 17, and 17). The approved protocol covered only participants aged 18 years or older. These three respondents were therefore excluded from all analyses and their records were deleted, leaving 57 age-eligible participants with complete baseline data. Of these, 27 provided complete retest data and 30 did not.

To examine potential attrition bias, retest completers were compared with non-completers on baseline EAT performance, age, and sex. Completers (*M* = −16.79, *SD* = 3.41) did not differ detectably from non-completers (*M* = −16.28, *SD* = 2.73) in baseline EAT performance (Welch’s test, *p* = .537) or in the distribution of baseline scores (Kolmogorov–Smirnov test, *p* = .553). Age also did not differ detectably between the groups, *p* = .309, nor did sex composition (Fisher’s exact *p* = .512). Thus, the observed attrition was not detectably associated with baseline emotional accuracy, age, or sex, although selection on unmeasured characteristics cannot be ruled out. The final analytic sample was 85.2% female (23/27) and ranged in age from 20 to 75 years (*M* = 35.48, *SD* = 15.26).

Participants were informed that the study consisted of two parts but were not told that the same videos would be presented again at Time 2. They were never shown the targets’ self-ratings or given accuracy feedback. Among participants in the final analytic sample, the elapsed time between Time 1 and Time 2 was *M* = 25.52 days (*SD* = 9.52, range = 12.49 to 55.39 days). The study was approved by the Institutional Review Board, and all participants provided informed consent.

#### 2.1.2. Study 2: Cross-Stimulus Consistency Design

Participants were undergraduate students who completed the EAT twice as part of course requirements. To test whether individual differences in emotional accuracy generalize across different naturalistic targets, a cross-stimulus consistency design was implemented, presenting different target sets at the two measurement occasions. At Time 1, participants watched and rated four videos, as at Time 2. Due to a technical problem in storing responses for one video, only three videos could be scored and included in the Time 1 EAT composite. A total of 67 students completed both sessions and consented to the use of their anonymized data for research purposes. The primary analytic sample was restricted to 62 participants (*N* = 62) who had complete data for all four Time 2 videos. This reflected an exclusion rate of 7.5% from the retested pool; the five participants who completed only two or three videos at Time 2 were retained for sensitivity analyses. The primary analytic sample (87.1% female) ranged in age from 20 to 46 years (*M* = 24.42, *SD* = 4.44). The elapsed time between sessions was *M* = 17.00 days (*SD* = 6.11, range = 7.14 to 40.82 days). The study was approved by the Institutional Review Board, and all participants provided informed consent.

### 2.2. Measures

**Figure 1:**
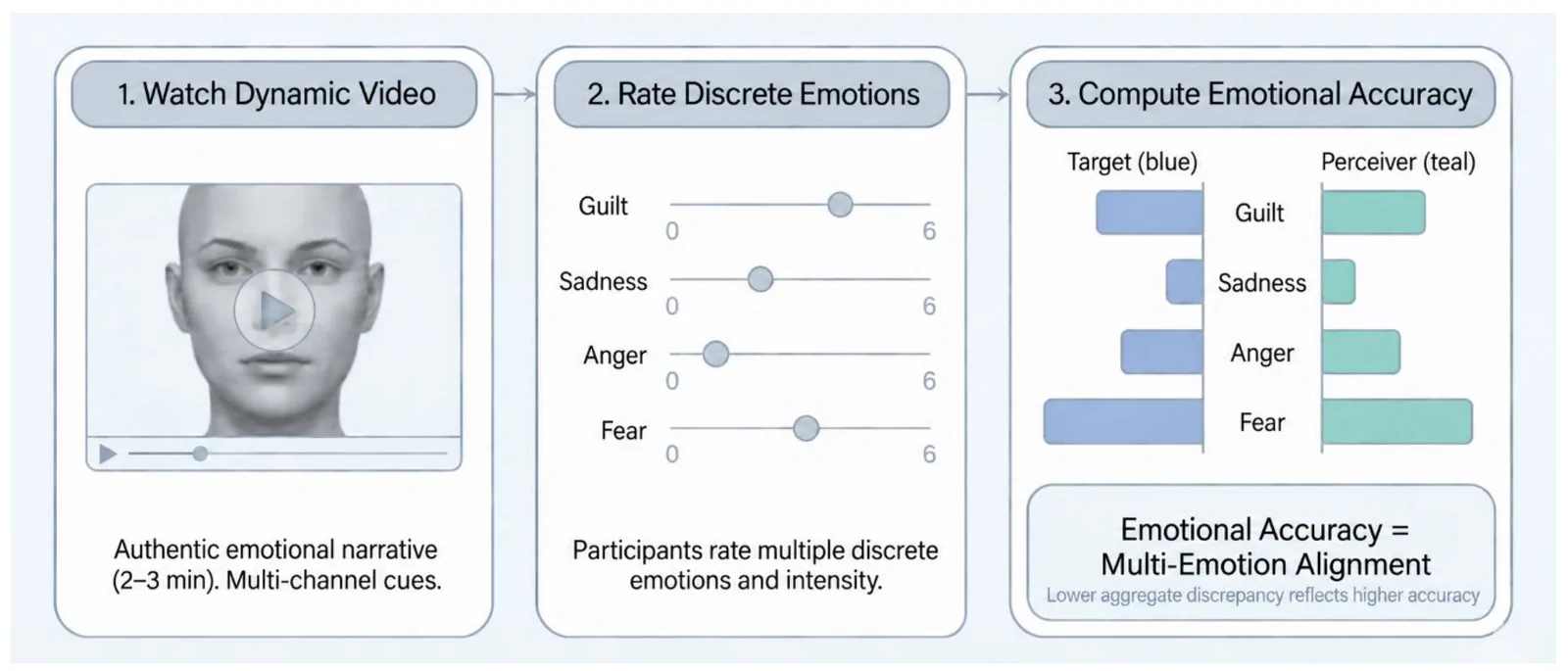
The Emotional Accuracy Test (EAT) paradigm. Participants watch dynamic narratives of authentic emotional events and rate multiple discrete emotions on graded intensity scales. Emotional accuracy is computed as the alignment between perceivers’ ratings and targets’ self-reported emotional experiences across emotions and intensities. Note. This schematic does not depict any participant or target from the study. A synthetic AI-generated face was used solely for illustration to avoid displaying identifiable target imagery and to protect participant privacy.

Emotional Accuracy Test (EAT). The Emotional Accuracy Test (EAT; Israelashvili et al., 2020) is a performance-based measure of emotional understanding that uses naturalistic interpersonal stimuli (see Figure 1 for illustration). The videos used in the present studies were selected from the original EAT stimulus pool. In each video, a target recounted a genuine emotionally significant event from their life. After the recording, targets viewed their own video and rated the intensity of 10 emotions experienced while recounting the event: surprise, sadness, guilt, anger, disappointment, worry, fear, embarrassment, confusion, and rage. Ratings were provided on a 7-point scale ranging from 0 (not at all) to 6 (very much) and were completed separately for each video. Participants watched the videos and used the same emotion labels and response scale to estimate the intensity of each emotion experienced by the target. Within each measurement occasion, all participants viewed the same target set, and video order was randomized. Study 1 presented the same four targets at both occasions. Study 2 used different target sets across occasions, with three scored videos at Time 1 (due to a response-storage problem) and four at Time 2. Emotional accuracy was calculated relative to each target’s self-reported emotional profile. For each video, the absolute discrepancy between the participant’s rating and the target’s self-rating was calculated separately for each of the 10 emotions and then summed. The overall EAT score was the negative mean of the video-level discrepancy totals. Scores could range from −60 to 0, with values closer to zero indicating greater emotional accuracy. McDonald’s omega (ω) was selected to evaluate internal consistency because naturalistic video stimuli naturally vary in emotional intensity and expressiveness, meaning they cannot be assumed to contribute equally to the overall score (Dunn et al., 2014). In Study 1, ω was .66, 95% CI [.38, .83], at Time 1 and .48, 95% CI [.18, .68], at Time 2. The paired bootstrap estimate of the change in omega (Time 2 minus Time 1) was −.18, 95% CI [−.45, .08]. Because the same four targets were presented at both occasions, target sampling cannot explain this difference. With *N* = 27, the observed decrease is compatible with sampling fluctuation and cannot be attributed to a specific mechanism from the present data. The Study 1 test–retest estimate should therefore be interpreted alongside the lower Time 2 internal consistency. In Study 2, ω was .76 at Time 1 and .74 at Time 2. These coefficients were treated as descriptive indicators of score coherence rather than definitive tests of unidimensionality.

### 2.3. Statistical Analysis

Analyses were conducted separately by study. Rank-order stability was estimated using Pearson’s *r* with Fisher-transformed 95% confidence intervals. Single-measure ICCs were estimated using two-way models (Shrout & Fleiss, 1979). ICC(A,1) quantified absolute agreement, whereas ICC(C,1) quantified consistency after systematic mean differences between sessions or forms were excluded from the disagreement term (Koo & Li, 2016; McGraw & Wong, 1996). In Study 1, ICC(A,1) was treated as the primary coefficient because the same target set was repeated and numerical reproducibility across comparable retest occasions was central. In Study 2, the two target sets were treated as specific, non-equated forms. ICC(C,1) was therefore treated as the primary estimate of consistency in participants’ relative standing, whereas ICC(A,1) assessed agreement between the raw scores produced by these two forms. ICC confidence intervals were estimated from 20,000 paired-participant percentile bootstrap samples. Cicchetti’s (1994) categories originated in clinical and inter-rater reliability contexts and are treated here as historical reference points. Koo and Li (2016) classify ICCs below .50 as poor, .50–.75 as moderate, and .75–.90 as good. More stringent guidance for individual-differences measurement has suggested that reliability below .70 is generally undesirable and values above .80 are preferable (Brysbaert, 2024). At the same time, reliability coefficients are continuous, and fixed thresholds should not be treated as universal pass/fail criteria (Parsons et al., 2019). Accordingly, the present coefficients are interpreted primarily through their point estimates, confidence intervals, and intended use.

Paired-samples *t* tests evaluated mean change (Time 2 minus Time 1), and *d*_z_ standardized that change by its *SD*. The standard error of measurement (SEM) was the square root of residual error variance plus the nonnegative systematic occasion variance component under the absolute-agreement model. The 95% smallest detectable difference was SDD_95_ = 1.96 × sqrt(2) × SEM. These descriptive estimates are conditional on the sampled occasions/forms; in Study 2 they incorporate form differences and should not be treated as calibrated individual-change thresholds. Table 1 reports all quantities. Study 1 used four scored videos and 40 emotion-by-video discrepancies at each occasion. Study 2 used three scored videos and 30 discrepancies at Time 1 and four scored videos and 40 discrepancies at Time 2, although four videos were administered at both occasions. A sensitivity analysis retained the five partial Time 2 composites. Exploratory relevance-based analyses are reported in Section S1.

Sensitivity analyses indexed general scale use as each participant’s mean emotion-rating intensity, computed separately at each occasion over the scored videos. EAT scores were regressed on occasion-specific mean intensity, and residuals were correlated across occasions. Age sensitivity analyses correlated age with EAT scores, estimated age-adjusted retest correlations, and restricted Study 1 to the Study 2 age range. Attrition comparisons contrasted retest completers and non-completers with age-eligible, complete baseline EAT data.

## 3. Results

### 3.1. Study 1: Repeated-Stimulus Reliability

**Figure 2:**
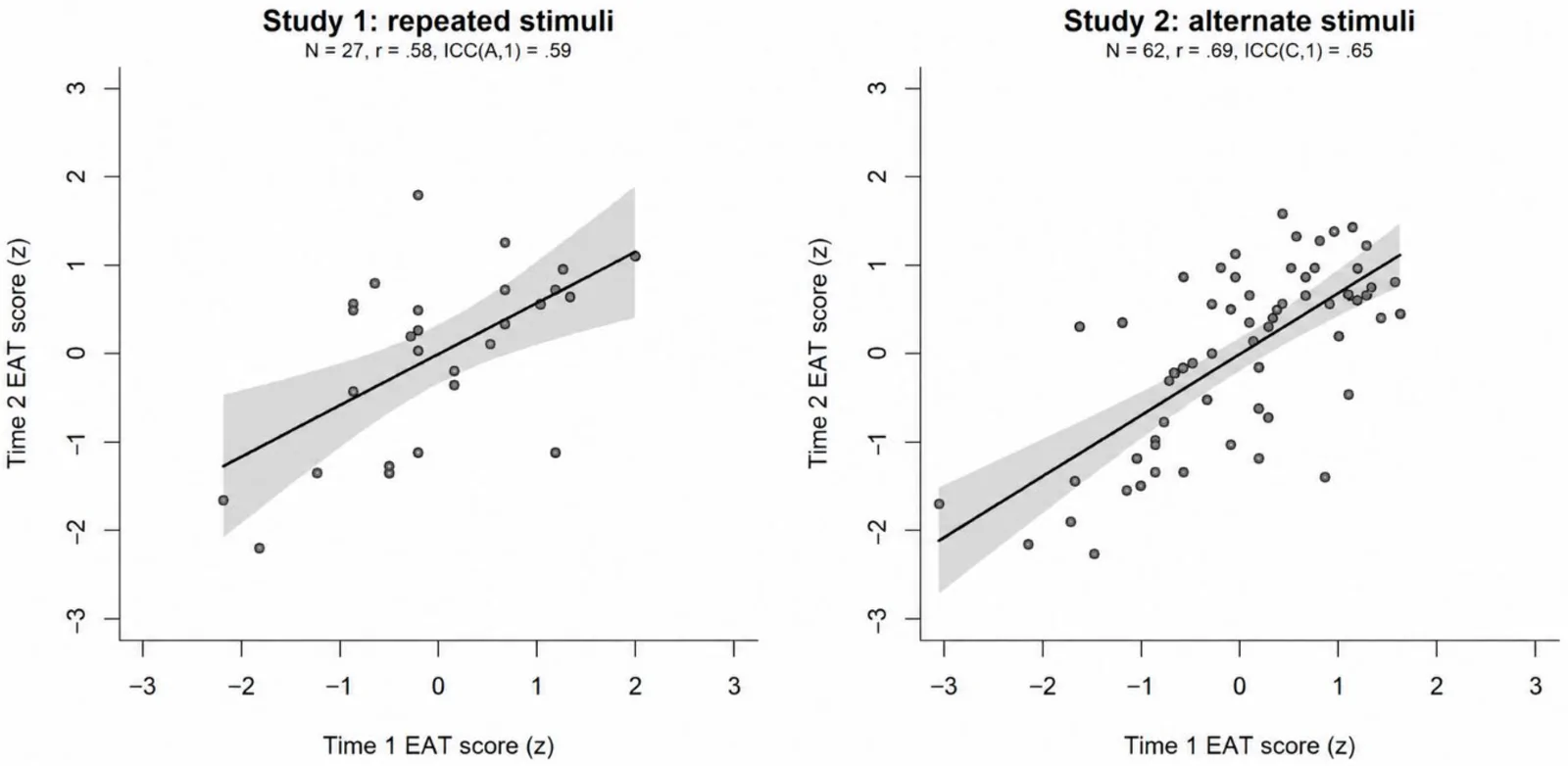
Temporal stability of Emotional Accuracy Test scores across repeated and alternate stimuli. Note. Emotional Accuracy scores are standardized within occasion for display. Solid black lines represent linear regression fits with 95% confidence interval bands. Standardization removes mean differences; Figure S2 shows raw scores on identical axes.

In Study 1 (*N* = 27), scores were *M* = −16.79 (*SD* = 3.41) at Time 1 and *M* = −17.10 (*SD* = 3.26) at Time 2. Rank-order stability was *r* = .58, 95% CI [.26, .79], and absolute agreement was ICC(A,1) = .59, 95% CI [.20, .80] (Table 1; Figure 2). Consistency was ICC(C,1) = .58, 95% CI [.20, .81]. The wide confidence interval indicates substantial uncertainty regarding the magnitude of temporal stability. There was no evidence of systematic mean-level change across occasions, *t*(26) = −0.53, *p* = .598, *d*_z_ = −0.10 (mean change = −0.31). Figure S1 presents individual trajectories for the final analytic samples.

### 3.2. Study 2: Alternate-Stimulus Reliability

In Study 2 (*N* = 62), perceivers evaluated different target sets across the two occasions. EAT performance maintained rank-order stability (*r* = .69, 95% CI [.53, .80], *p* < .001; Figure 2) and consistency reliability (ICC(C,1) = .65, 95% CI [.49, .76]), indicating that participants preserved their relative standing across distinct targets (see Table 1). However, absolute agreement was lower (ICC(A,1) = .52, 95% CI [.37, .63]), with higher accuracy scores at Time 2 (*M* = −18.97, *SD* = 4.88) compared to Time 1 (*M* = −23.33, *SD* = 6.99; *t*(61) = 6.79, *p* < .001, *d*_z_ = 0.86; Figure S1). A rank-based non-parametric analysis yielded a similar association (Spearman’s ρ = .67, *p* < .001). Sensitivity analyses retaining participants with partial Time 2 data (*N* = 67) confirmed this overall pattern of results (see Table 1). Raw-score associations plotted on identical axes are provided in Figure S2 in the Supplementary Materials.

### 3.3. Sensitivity Analyses: Mean Rating Intensity and Age

To examine whether the primary reliability estimates were sensitive to stable response-scale use or age differences, supplementary sensitivity analyses were conducted across both studies.

Mean rating intensity was temporally stable in Study 1 (*r* = .75) and Study 2 (*r* = .71, *p* < .001). After EAT scores were residualized for occasion-specific mean rating intensity, the Study 1 test–retest correlation was *r* = .65, 95% CI [.36, .83], *p* < .001, compared with the unadjusted *r* = .58. In Study 2, the corresponding association decreased from *r* = .69 to *r* = .49, 95% CI [.27, .66], *p* < .001. Thus, adjustment for general scale use did not reduce temporal stability in Study 1 but attenuated, without eliminating, the cross-stimulus association in Study 2.

In Study 1, age was weakly associated with Time 1 EAT (*r* = −.05, *p* = .800) and Time 2 EAT (*r* = .02, *p* = .927); the age-adjusted retest correlation remained approximately .58. Restricting Study 1 to ages 20 to 46 years (*N* = 20) yielded *r* = .62 and ICC(A,1) = .63. In Study 2, age correlations were *r* = .09 (*p* = .503) and *r* = .11 (*p* = .382) at Times 1 and 2, respectively, and the age-adjusted retest correlation was approximately .69. Thus, age did not materially account for the observed reliability estimates in either study.

Exploratory category-level analyses detailing how temporal stability varies across core, secondary, and absent emotional states are presented in Section S1.

## 4. Discussion

The present research examined whether the Emotional Accuracy Test (EAT) preserves meaningful individual differences across repeated administrations. Two complementary retest designs were used. In Study 1, participants evaluated the same four targets at both sessions. Rank-order stability was *r* = .58, 95% CI [.26, .79], and absolute agreement was ICC(A,1) = .59, 95% CI [.20, .80], over a mean interval of 25.52 days, with no evidence of systematic mean-level change. These findings are consistent with temporal stability, although the estimate was imprecise. In Study 2, participants evaluated different target sets at two sessions. They retained their relative standing across different stimulus sets, with rank-order stability of *r* = .69, 95% CI [.53, .80], and consistency of ICC(C,1) = .65, 95% CI [.49, .76]. Absolute agreement was lower, ICC(A,1) = .52, 95% CI [.37, .63], with higher accuracy scores at the second session. Taken together, the findings provide preliminary evidence that EAT performance preserves individual differences that are expressed across time and across different target sets. At the same time, the contrast between the studies demonstrates that stability in relative standing does not imply that scores obtained from different stimulus sets are numerically interchangeable.

The observed coefficients fall within the broad range reported for other behavioral measures of social cognition, but differences in design, samples, and precision limit direct comparison. Ferguson and Inzlicht (2023), for example, reported ICCs of .65 and .67 for the Empathy Selection Task. More broadly, Pennington et al. (2025) found considerable variation across commonly used social-cognitive tasks, with ICCs ranging from poor to good depending on the task and scoring index. Such variability is consistent with broader concerns that tasks capable of producing robust experimental effects do not necessarily provide reliable measures of individual differences (Hedge et al., 2018; Parsons et al., 2019). In the closely related continuous valence empathic accuracy paradigm, Kern et al. (2013) reported four-week retest correlations of .67 and .72 for scores based on six and nine videos, respectively. These estimates should be compared cautiously with the present findings: Kern et al. assessed rank-order correlations rather than absolute agreement, included more targets, and studied a heterogeneous clinical sample in which greater between-person variability may have facilitated reliability. The present research complements this evidence by evaluating a multidimensional emotional-profile measure in two nonclinical samples and by explicitly distinguishing the reproduction of raw scores from the preservation of participants’ relative standing.

These findings also reinforce the principle that reliability belongs to a particular score obtained under particular measurement conditions, rather than to a task in the abstract (Parsons et al., 2019; Vacha-Haase, 1998). In the present studies, reliability estimates differed depending on whether the focus was relative stability, consistency, or absolute agreement. Study 2 further illustrates this score specificity: the same scoring approach preserved participants’ relative standing across target sets, whereas absolute agreement was lower because raw score levels differed across occasions. Consequently, researchers should report the scoring rule, target-set composition, number of scored videos, completeness criterion, and reliability definition relevant to their inferences.

This distinction is particularly important when interpreting absolute-agreement coefficients. Under the fitted two-way absolute-agreement model, ICC(A,1) expresses between-person variance relative to total variance that also includes systematic occasion effects and residual error. It should therefore not be interpreted as a model-free estimate of stable trait variance. The alternate-stimulus findings provide complementary evidence for stable individual differences in EAT performance: participants who were relatively accurate when judging one set of targets also tended to be relatively accurate when judging another set. This pattern suggests that EAT performance is not determined solely by target-specific knowledge or idiosyncratic compatibility between a particular perceiver and target. Exploratory analyses in Section S1 further suggest that temporal stability may vary with the emotional relevance of the rated states, pointing to the potential importance of the specific emotional content sampled. At the same time, the response-style sensitivity analysis qualifies this interpretation. Adjustment for mean rating intensity did not reduce the Study 1 association, whereas the Study 2 correlation decreased from .69 to .49. Mean rating intensity is not exclusively a response-style index: because the target profiles include many low-intensity emotions, a perceiver’s characteristic rating intensity may also capture genuine accuracy-relevant variance. Residualizing mean intensity may therefore remove part of the signal as well as scale-use variance. The adjusted *r* = .49 is best interpreted as a conservative sensitivity estimate rather than a uniquely corrected estimate. Importantly, a cross-stimulus association remained after this adjustment.

The mean-level findings across studies further delineate the boundaries of score stability. In Study 1, repeated presentation of the same targets was not associated with detectable systematic mean-level change in this sample, although an exposure effect cannot be ruled out. In Study 2, participants maintained their relative standing across target sets (ICC(C,1) = .65), but raw scores were higher at Time 2, resulting in lower absolute agreement (ICC(A,1) = .52). Crucially, this mean shift should not be interpreted as evidence of learning or practice. Because target set was confounded with occasion, the scored composites differed in length (three videos at Time 1 vs. four at Time 2), and the forms were not counterbalanced, the higher Time 2 scores cannot be uniquely attributed to occasion, target-set differences, or scored form length. Thus, perceivers’ relative standing generalized across targets, whereas raw scores remained stimulus-dependent.

Together, these findings inform the broader conceptual debate regarding whether emotional understanding reflects a stable individual difference, a malleable skill, or both. The present studies provide evidence concerning stability, showing that perceivers preserve relative standing across time and, to some extent, across different targets. Evidence for malleability, however, comes from prior feedback and training studies, which suggest that emotional accuracy can improve following structured training and targeted feedback (Israelashvili & Perry, 2021; Israelashvili et al., 2025). Viewed alongside prior intervention evidence, the present stability findings are consistent with emotional accuracy as a relatively stable yet learnable skill. This combination supports the use of the EAT for examining baseline individual differences and group-level mean change following training or intervention. The reported SDD_95_ values (6.00 in Study 1 and 13.02 in Study 2) do not support interpreting individual change scores as reliable improvement or deterioration.

## 5. Limitations and Future Directions

Several limitations of the current research should be acknowledged. First, Study 1 included a relatively small sample. Although the point estimates remain informative, the modest sample produced wider confidence intervals around the variance components underlying the ICC. Future studies with larger samples are therefore needed to estimate reliability with greater precision (Bonett, 2002; Williams et al., 2025). Attrition represents a separate limitation. Comparisons of age-eligible participants with complete baseline EAT data did not reveal detectable differences between retest completers and non-completers in baseline performance, score distribution, age, or sex, but selective retention on unmeasured characteristics cannot be ruled out. The predominantly female samples and convenience- and course-linked recruitment procedures also limit population-level generalizability. Although Study 2 included a larger sample, future research should examine stability across more diverse clinical and nonclinical populations to determine whether EAT reliability depends on the range and heterogeneity of emotional-understanding ability in the sampled population.

A further limitation concerns the repeated-stimulus design of Study 1. Participants were never shown the targets’ self-ratings or given accuracy feedback, so memory for a supplied correct criterion response was not possible. However, recognition of the videos or recall of one’s own prior judgments cannot be ruled out because neither was directly measured.

Study 2 introduced a different design limitation. Target set and occasion were confounded. Four videos were watched and rated at both occasions, but a response-storage problem left three scored videos at Time 1 versus four at Time 2. The unequal number of scored videos may have affected the cross-stimulus estimates. Although shorter measures can in principle be less reliable, the three-video Time 1 composite did not show lower internal consistency than the four-video Time 2 composite (ω = .76 vs. .74). Given the heterogeneous naturalistic stimuli, the extent to which unequal scored form length affected the observed association cannot be determined from the present data. Future research using equal-length, counterbalanced forms is needed to separate occasion effects from target-set differences.

Across both studies, the limited breadth of target sampling remains an important consideration (Westfall et al., 2015). Both studies relied on a small number of target videos featuring primarily negative emotional experiences. In Study 1, internal consistency was lower at Time 2 (ω = .48), but the same targets were used at both occasions and the paired bootstrap confidence interval for the change in omega included zero. Thus, the present data do not establish a reliable between-occasion decrease or identify its source. Lower within-occasion coherence should nevertheless be considered when interpreting the observed temporal association. Because EAT videos deliberately sample heterogeneous targets, narratives, and expressive styles rather than functioning as interchangeable items, future research should examine whether increasing target quantity and diversity improves score dependability and generalizability. Future research could also extend classical test–retest analysis using Generalizability Theory or multilevel variance-decomposition designs that separate perceiver, target, emotion, occasion, and interaction components (Judd et al., 2012; Shavelson et al., 1989). Such designs could help determine how many targets and emotion ratings are required for dependable individual-difference scores.

## 6. Conclusions

The present findings provide additional psychometric support for the EAT in group-level and correlational research, particularly when measurement error is explicitly considered. Study 1 supports temporal stability, although the precision of the estimate was limited, and Study 2 indicates preservation of relative standing across different targets despite differences in raw score levels. Together, the complementary designs provide converging evidence that individual differences in EAT performance show reproducibility across time and, to some extent, across different target sets. These findings should be interpreted in light of the limited samples, target sampling, response-style sensitivity, and non-equated scored forms. Accordingly, the present evidence does not provide sufficient support for the use of the EAT in individual-level assessment or diagnostic classification. Within these bounds, however, the findings strengthen the empirical basis for using the EAT to investigate individual differences in emotional accuracy in group-level and correlational research.

## Figures and Tables

**Table 1:** Test–retest reliability metrics, intraclass correlations, and mean-level changes across studies.

Analysis	*N*	T1 *M* (*SD*)	T2 *M* (*SD*)	*r* [95% CI]	ICC(A,1) [95% CI]	ICC(C,1) [95% CI]	Change T2 − T1	*p*	SEM	SDD_95_
Study 1	27	−16.79 (3.41)	−17.10 (3.26)	.58 [.26, .79]	.59 [.20, .80]	.58 [.20, .81]	−0.31	.598	2.17	6.00
Study 2 complete	62	−23.33 (6.99)	−18.97 (4.88)	.69 [.53, .80]	.52 [.37, .63]	.65 [.49, .76]	4.36	<.001	4.70	13.02
Study 2 sensitivity	67	−23.59 (7.16)	−19.16 (5.33)	.72 [.58, .82]	.56 [.41, .68]	.69 [.54, .80]	4.44	<.001	4.68	12.97

Note. Mean change is calculated as Time 2 minus Time 1 (values closer to zero indicate greater accuracy). CI = 95% confidence interval; ICC(A,1) = absolute agreement; ICC(C,1) = consistency; SEM = standard error of measurement; SDD_95_ = 95% smallest detectable difference. ICC intervals are percentile bootstrap intervals. SDD_95_ = 1.96 × sqrt(2) × SEM. Study 2 primary analyses require complete data across all four Time 2 videos (*N* = 62); sensitivity analyses retain five participants with partial retest composites (*N* = 67). SEM and SDD_95_ are descriptive estimates conditional on the sampled occasions/forms, not validated individual-change thresholds.

## Data Availability

The data supporting the findings of this study are available from the author upon reasonable request.

## Supplementary Materials

The following supporting information can be downloaded at https://www.mdpi.com/article/10.3390/jintelligence14100240/s1. Figure S1. Individual-level trajectories of Emotional Accuracy scores across measurement occasions; Figure S2. Raw-score associations between Time 1 and Time 2 Emotional Accuracy scores; Section S1. Exploratory Decomposition of Emotional Accuracy by Target-Rated Emotion Relevance, including Table S1.
