# Supplementary material for: Stable Across Time and Targets? Test–Retest Reliability of the Emotional Accuracy Test
Source: J Intell. 2026 Oct 2;14(10):240. doi: 10.3390/jintelligence14100240 (PMC13705777; doi:10.3390/jintelligence14100240)
Supplement: Supplementary file 1 [file jintelligence-14-00240-s001.zip › jintelligence-4505583-supplementary.pdf]

Section S1

Exploratory Decomposition of Emotional Accuracy by Target-Rated Emotion Relevance

Analytic Approach: Exploratory relevance-based scores were computed in both studies to examine whether temporal stability differed according to the intensity with which each emotion was experienced by the target. Emotion-by-video observations were classified according to the target's criterion rating as absent (0), secondary (1 to 3), or core (4 to 6). For each category, emotional accuracy was calculated as the negative mean absolute discrepancy between the participant's rating and the target's self-rating across all emotion-by-video observations assigned to that category. Values closer to zero indicated greater accuracy. Because the number of observations within each category (i.e., absent, secondary, or core) differed across stimulus sets, category scores were averaged over all available observations in that category rather than summed. In Study 1, category-level reliability was evaluated using Pearson's r and $\mathrm{ICC}(A, 1)$ because the same targets were presented at both sessions. In Study 2, both $\mathrm{ICC}(C, 1)$ and $\mathrm{ICC}(A, 1)$ were calculated to distinguish the preservation of participants' relative standing from the equivalence of raw scores obtained from the two target sets. Paired-samples t-tests were used to assess mean change within each category.

A sensitivity analysis applied an alternative classification in which target ratings of 0 to 1 were defined as low-intensity or minimally present emotions, ratings of 2 to 3 as secondary emotions, and ratings of 4 to 6 as core emotions. Because this alternative classification produced sparse and unevenly distributed secondary-emotion scores, particularly at Time 1 in Study 2, it was treated as a robustness check rather than as a primary specification. Under the primary classification, Study 1 included 14 absent, 13 secondary, and 13 core emotion-by-video observations at each session. Study 2 included 16 absent, 9 secondary, and 5 core observations at Time 1, and 14 absent, 13 secondary, and 13 core observations at Time 2. Under the alternative classification, the corresponding counts were 20, 7, and 13 in Study 1; 21, 4, and 5 at Study 2 Time 1; and 20, 7, and 13 at Study 2 Time 2.

Results

Study 1: Repeated-Stimulus Reliability

In the final Study 1 adult-only sample (N = 27), accuracy concerning core emotions yielded r = .70, ICC(C,1) = .69, and ICC(A,1) = .70. For absent emotions, r = .64, ICC(C,1) = .64, and ICC(A,1) = .63. For secondary emotions, r = .46, ICC(C,1) = .46, and ICC(A,1) = .43. Mean-change p values were .741, .220, and .066 for core, absent, and secondary emotions, respectively. These exploratory point estimates suggest differing stability across relevance categories, without establishing distinct subscales.

Under the alternative classification, low/minimally present emotions yielded r = .63, ICC(C,1) = .63, and ICC(A,1) = .63. Secondary emotions yielded r = .50, ICC(C,1) = .49, and ICC(A,1) = .49; core emotions yielded r = .70, ICC(C,1) = .69, and ICC(A,1) = .70. The alternative secondary score contained only seven emotion-by-video observations and was not represented in every video; the pattern is interpreted descriptively.

Study 2: Alternate-Stimulus Reliability

In Study 2 ($N = 62$), accuracy for absent emotions yielded $r = .71$, ICC(C,1) = .71, and ICC(A,1) = .72. There was no evidence of systematic mean change for absent emotions ($p = .636$). Accuracy for secondary emotions yielded $r = .61$, ICC(C,1) = .59, and ICC(A,1) = .45; core emotions yielded $r = .51$, ICC(C,1) = .50, and ICC(A,1) = .41. Both scores were higher at Time 2 (both *p*s < .001). The difference between forms was concentrated in emotions targets experienced to some degree, rather than those they rated as absent.

The alternative classification yielded a similar pattern. For low-intensity emotions, consistency and absolute agreement were ICC(C,1) = .71 and ICC(A,1) = .69, respectively. The corresponding coefficients were .53 and .51 for secondary emotions, and .50 and .41 for core emotions. However, the alternative secondary score was based on only four observations at Time 1 and seven observations at Time 2. These estimates should therefore be interpreted cautiously. Table A1 summarizes the primary-classification estimates for both studies.

Table S1. *Reliability of Emotional Accuracy by target-rated emotion relevance.*

Study	**Category**	**Pearson's *r***	**ICC(C,1)**	**ICC(A,1)**	**Mean-change *p***
Study 1	Absent, 0	.64	.64	.63	.220
Study 1	Secondary, 1 to 3	.46	.46	.43	.066
Study 1	Core, 4 to 6	.70	.69	.70	.741
Study 2	Absent, 0	.71	.71	.72	.636
Study 2	Secondary, 1 to 3	.61	.59	.45	< .001
Study 2	Core, 4 to 6	.51	.50	.41	< .001

Note: Study 1 $N = 27$; Study 2 $N = 62$. Category scores are negative mean absolute discrepancies per emotion-by-video observation, unlike the main composite's negative mean total discrepancy per video. Values closer to zero indicate greater accuracy. ICC(C,1) quantifies consistency and ICC(A,1) absolute agreement. Study 1 repeated the same targets; Study 2 used different target sets.

Exploratory interpretation

The relevance-based analyses showed a broadly convergent pattern across the two studies, with important qualifications. Accuracy for emotions that targets rated as absent showed relatively stable estimates in both the repeated-stimulus and cross-stimulus designs. This pattern may indicate that avoiding the attribution of emotions that a target did not experience reflects a relatively general perceiver tendency that transfers across targets. The point estimates for core emotions were higher when the same targets were repeated than when target sets changed. Accuracy for these emotions may therefore depend on the target, story, and expressive cues in each set. Secondary-emotion estimates were lower or

intermediate across coding specifications. These comparisons are exploratory and do not establish differences in population reliability.

These exploratory patterns should nevertheless be interpreted descriptively. Category scores depend not only on emotional discrimination but also on stable tendencies in how perceivers use the response scale. A general tendency to provide low ratings can increase apparent accuracy for absent or low-intensity emotions, whereas a tendency to use higher response values may favor accuracy for core emotions. In addition, the categories contained unequal and sometimes very small numbers of observations, particularly for secondary and core emotions in the first form of Study 2. The analyses therefore do not establish distinct EAT subscales. Rather, they identify potentially informative error profiles that warrant examination with larger, balanced, and psychometrically calibrated stimulus sets.

Supplementary Figures for the Main Analyses

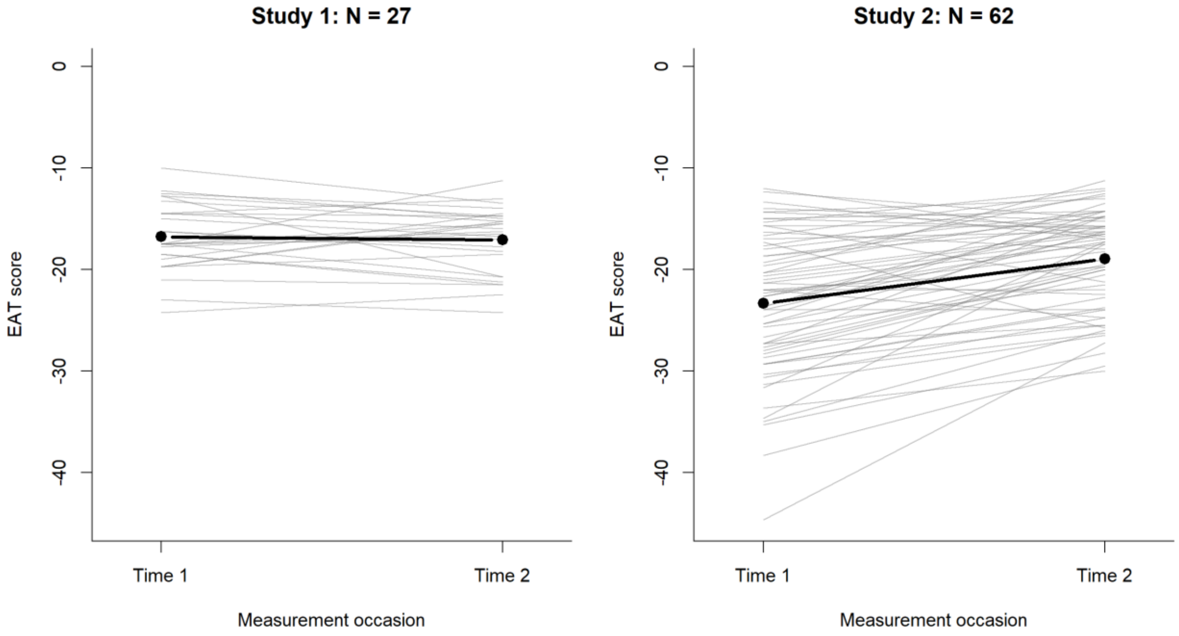

Figure S1*. Individual-level trajectories of Emotional Accuracy scores across measurement occasions. Study* 1*: final adult-only sample (N* = 27*). Study* 2*: primary sample (N* = 62*).*

Note: Each thin line represents an individual participant's trajectory from Time 1 to Time 2. The bold line represents the group mean trajectory. All scores reflect negative mean total absolute discrepancies per scored video, such that values closer to zero indicate greater accuracy.

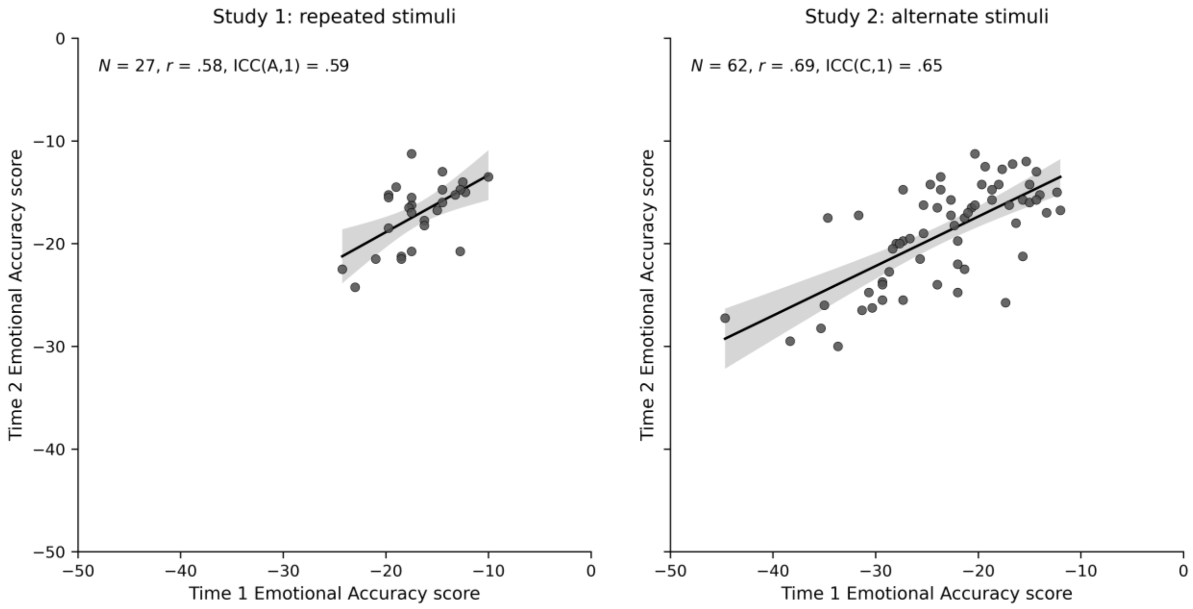

Figure S2. *Raw-score associations between Time* 1 *and Time* 2 *Emotional Accuracy scores. Note: Axes use the same raw-score limits in both panels; higher values indicate greater accuracy. Solid lines are linear regression fits and shaded bands are* 95% *confidence intervals.*
